# Mobile Health Adoption in Mental Health: User Experience of a Mobile Health App for Patients With an Eating Disorder

**DOI:** 10.2196/12920

**Published:** 2019-05-31

**Authors:** Dimitra Anastasiadou, Frans Folkvord, Eduardo Serrano-Troncoso, Francisco Lupiañez-Villanueva

**Affiliations:** 1 Department of Information and Communication Sciences Universitat Oberta de Catalunya Barcelona Spain; 2 Open Evidence Research Barcelona Spain; 3 School of Humanities and Digital Sciences Tilburg University Tilburg Netherlands; 4 Child and Adolescent Psychiatry and Psychology Department, Hospital Sant Joan de Déu of Barcelona Barcelona Spain; 5 Children and Adolescent Mental Health Research Group, Institut de Recerca Sant Joan de Déu Barcelona Spain

**Keywords:** eating disorders, mental health, mHealth, mobile applications, focus groups

## Abstract

**Background:**

Despite the worldwide growth in mobile health (mHealth) tools and the possible benefits for both patients and health care providers, the overall adoption levels of mHealth tools by health professionals remain relatively low.

**Objective:**

This study aimed (1) to investigate attitudes of health care providers and mHealth experts toward mHealth tools in the health context in general, and this study aimed (2) to test the acceptability and feasibility of a specific mHealth tool for patients with an eating disorder (ED), called TCApp, among patients and ED specialists.

**Methods:**

To this purpose, we conducted an explorative qualitative study with 4 in-depth group discussions with several groups of stakeholders: our first focus group was conducted with 11 experts on mHealth from the Catalan Association of Health Entities; the second focus group included 10 health care professionals from the Spanish College of Doctors of Barcelona; the third focus group involved 9 patients with an ED who had used the TCApp over a 12-week period, and the fourth and last focus group involved 8 ED specialists who had monitored such ED patients on the Web.

**Results:**

The focus groups showed that health care providers and mHealth experts reported barriers for mHealth adoption more often than facilitators, indicating that mHealth techniques are difficult to obtain and use. Most barriers were attributed to external factors relating to the human or organizational environment (ie, lack of time because of workload, lack of direct interest on a legislative or political level) rather than being attributed to internal factors relating to individual obstacles. The results of the mHealth intervention study indicate that the TCApp was considered as easy to use and useful, although patients and the ED specialists monitoring them on the Web reported different adoption problems, such as the inability to personalize the app, a lack of motivational and interactive components, or difficulties in adhering to the study protocol.

**Conclusions:**

In general, this paper indicates that both health professionals and patients foresee difficulties that need to be addressed before comprehensive adoption and usage of mHealth techniques can be effectively implemented. Such findings are in line with previous studies, suggesting that although they acknowledge their possible benefits and cost-effectiveness, health care providers are quite resistant and conservative about integrating mHealth technologies in their daily practice.

## Introduction

### Background

Over the last decade, the number of people who have obtained a mobile phone or some other portable electronic communication devices worldwide has increased exponentially [1]. As a consequence, innovative apps for such devices have been developed to address health issues, and this has evolved into a new field labeled as mobile health (mHealth) [2,3]. Central to the concept of mHealth is that by removing geographical, temporal, and other barriers that influence usage of interventions that are related to health behavior, information and resource services are more flexible and have the potential to reach anyone, anytime, and anywhere [4].

mHealth has many different applications, such as the facilitation of data collection, the encouragement of health care consumers to adopt healthy lifestyles, the self-management of chronic conditions, such as pain, psychological distress, fatigue, and sleep [5,6], and the improvement of the delivery of health care services by targeting health care providers or by easing communication between these providers and their patients [7,8]. Despite the reported benefits for the patient and the health care provider, as well as mHealth’s positive impact on the economic and organizational delivery systems [9], the overall adoption of mHealth by health professionals remains relatively low [10].

Many factors influencing the acceptance, adoption, and usage of mHealth by health care professionals have been reported in the systematic review by Gagnon and colleagues [11]. Specifically, factors relating to mHealth characteristics, such as perceived usefulness and ease of use of the tool, have been identified as the main positive drivers for mHealth adoption by health professionals. In addition, individual-level factors are also considered decisive when assessing adoption levels, such as individuals’ time availability (eg, overcoming disruption of workflow), a positive risk-benefit assessment, and the level of confidence in and agreement with mHealth implementation.

Meanwhile, important barriers relating to mHealth characteristics were also mentioned in the same review [11], such as security, privacy, and cost issues, as well as design and technical concerns, in addition to factors relating to the human environment (eg, patient-health professional interaction) and other factors relating to the organizational environment, for example, workload, time constraints, dysfunctional relations among colleagues, and lack of human resources to support Information and Communication Technologies (ICT). Next to that, lack of integration and interoperability and the need for high-risk investment in unsure markets still present major barriers for adoption in the international literature [12]. Last but not least, service availability (ie, lack of connectivity) has also been identified as both a driver and a barrier for adoption by health care professionals [12,13].

Owing to the advantages of the use of mHealth technologies, it is important to determine what types of drivers and barriers are considered relevant by health professionals to adopt mHealth technologies efficiently. In this study, we will focus on the drivers and barriers for adoption of mHealth techniques in general health care delivery processes, and, more specifically, in an intervention using an mHealth tool specifically designed for patients with eating disorders (EDs) and the health care providers treating them.

### Mobile Health Adoption in Mental Health: An Experience With Patients With Eating Disorders

According to Holmes and colleagues [14], mHealth can transform the availability and efficacy of psychological treatments for mental health problems, given that individual therapy in the past few decades has not been able to meet the increasing demand for psychological services [15]. More specifically, the use of app-based treatments, both self-delivered and therapist-led, has the potential to improve traditional psychotherapy by facilitating monitoring of symptoms and outcomes, improving access to psychoeducation materials and skills training, and offering patients the opportunity to communicate with their therapist [16]. Other functionalities of apps include personalization, setting reminders, and the real-time collection and visual presentation of data, which could offer tremendous added value to traditional, *static* interventions. Correspondingly, *blended treatment*, referring to face-to-face treatments, which include a digital intervention or component, has been gaining in popularity over the last few years [17-19], and it has shown promising results in various mental health treatments [20,21].

Health professionals treating patients with EDs might be an important group that could benefit from mHealth interventions, either as an adjunct to standard treatment or as a method to providing existing evidence-based treatments on the Web [22]. According to National Institute for Health and Care Excellence guidelines [23], the gold-standard psychological therapy for adult patients with EDs is cognitive behavioral therapy (CBT) [24], which considers self-monitoring of dietary intake and associated thoughts and feelings as one of its main behavioral components [25]. Self-monitoring is a task that can easily be implemented through real-time self-records via mHealth techniques [26]. Literature has shown that both users and clinicians find mobile phone apps that support and facilitate ED symptom monitoring to be highly practicable as a component of ED treatment [27,28]. However, somewhat contradictory findings were reported by a recent study in which the advantages and disadvantages of the implementation of an mHealth tool in ED treatment were discussed [29,30]. Despite the enthusiasm surrounding mHealth technologies [31] and the fact that they may address limitations of face-to-face treatments (low motivation to practice, generalize and maintain therapeutic skills, and limited accessibility) [32], the necessity to develop guidelines for correct patient and clinician app usage is apparent [29,30].

Furthermore, factors that facilitate or hinder the effective adoption of these tools by patients and clinicians need to be identified through qualitative research. In a systematic review of the literature by Anastasiadou and colleagues [22] that focused on mHealth interventions for EDs, qualitative analyses showed that although most mHealth interventions were considered as acceptable, easy to use, and motivating by patients and therapists, other individual studies highlighted various problems surrounding their use, such as high dropout rates, technical issues, and privacy and personalization concerns [27,28]. Work by Juarascio and colleagues [32] confirmed that the limited number of acceptability and feasibility studies in this emergent field prevents researchers from drawing firm conclusions to date. Finally, the complexity of EDs, involving low treatment adherence and the need for an intensive level of care, may not make mHealth interventions the most suitable treatment in all cases. To address such matters, the 2 objectives of this study were (1) to investigate attitudes of health care providers and mHealth experts regarding mHealth tools in the health care context in general and (2) test the acceptability and feasibility of a specific mHealth tool designed specifically as an adjunct to standard treatment for patients with an ED and their therapists. To explore these 2 objectives, we conducted an explorative qualitative study with in-depth group discussions with various groups of stakeholders.

## Methods

### Design

We conducted 4 separate in-depth focus groups for this study. The first was carried out with health care providers, and the second was carried out with mHealth experts. Both focus groups had the same structure, and both aimed at assessing important drivers and barriers, which these professionals perceived relating to mHealth adoption in different health care services. The third and fourth focus groups were based on data from a larger randomized controlled trial (RCT) assessing the effectiveness of an mHealth intervention (ClinicalTrials.gov Identifier: NCT03197519), and they sought to assess the user (patient and provider) experience of a specific mHealth tool, called TCApp, in supporting self-management for patients with an ED. Focus group discussions were performed in accordance with the generic inductive approach [33].

### The TCApp

The TCApp is an mHealth tool specifically designed to bidirectionally connect patients with EDs and their therapists in the periods between medical consultations. The app has been designed and developed by the Spanish start-up company HealthApp. It offers 4 different language options (Spanish, Catalan, French, and English), and it is available through both Google Play and Apple Store markets, and it currently has more than 1000 patients as active users. Through the TCApp, patients can record their thoughts, actions, emotions, and whatever other information their therapists consider relevant for the therapy. The app can be customized for each patient according to therapy requirements. The main objective of the app is to replace paper self-records with Web-based records, as the former sometimes present a source of discomfort for patients [34]. In addition, the app also introduces gamification esthetics [35], including prizes, rewards, and reminders to improve patients’ engagement. Finally, it includes a chat function to facilitate patient-therapist communication between their regular face-to-face sessions. To date, the app has been used by over 1000 patients, counting with the special guidance of over 80 registered clinicians who are currently using the app’s clinician interface across 20 different centers in Spain.

### Study Sample

#### Health Care Providers and Mobile Health Experts

A total of 11 experts on mHealth from the Catalan Association of Health Entities (Associació Catalana d‘Entitats de Salut, ACES) and 10 health care professionals from the Spanish College of Doctors of Barcelona (Colegio Oficial de Médicos de Barcelona, COMB) with a special interest in mHealth were invited to participate in focus groups organized by our research group at the Open University of Catalonia (Universitat Oberta de Catalunya, UOC). The generic inductive approach, on which the design of the study was based, required a purposive sampling. To better identify barriers and facilitators for mHealth adoption from the point of view of 2 different stakeholders’ groups, we considered it important to carry out the first focus group with experts on mHealth from ACES and the second with health care professionals from COMB. The approval of the Ethical Committee of the University leading the study (UOC) to conduct the focus groups was obtained on February 21, 2017. Inclusion criteria for both focus groups were professionals from several private medical centers in Barcelona (Spain) who were actively engaging in mHealth technique usage, including both health care providers as well as mHealth experts, such as technical staff (eg, data analysts, software developers), ICT management staff, marketing and communication managers, and human resources management staff.

#### Participants of the Mobile Health Intervention using the TCApp

The study’s population comprised a larger RCT for the mHealth intervention study, involving 8 public and private ED units in Spain. A total of 108 patients were recruited from this larger study, the protocol of which has been published elsewhere [36]. It is worth mentioning that the Institutional Review Board approval was obtained for each participating institution and for the University leading the study (UOC). Participants were selected from this larger RCT, and they were invited to take part in our focus groups. Inclusion criteria for the patients included (1) having been part of the experimental group and used the TCApp for at least 8 out of the 12 weeks in which the app was available to participants and (2) not presenting any significant symptom worsening or showing relapse at the time in which the invitation was sent.

Inclusion criteria for the ED specialists’ group included having monitored their patients on the Web and having performed the following actions at least once a week: follow the patient’s daily self-records and generate personalized reports or graphs and communicate with him or her via the chat function. From a total of 21 patients who were invited to the focus group, 11 decided to participate, whereas 2 had to be excluded, as they did not meet all inclusion criteria. Next to that, all invited ED specialists from the 8 ED units took part in their respective group discussion. However, it is worth mentioning that only patients from 3 out of the 8 ED units involved in the larger study participated in the focus group study. The final focus group sample thus comprised 9 patients with an ED and 8 specialists who were monitoring such patients on the Web through the TCApp. Tables 1 and 2 show the clinical and demographic characteristics of the patients and ED specialists at baseline.

**Table 1:** Description of patients.

Patient characteristics (*N*=9)		Values
Age (years), mean (SD)		15 (0.50)
Body Mass Index (actual), mean (SD)		19.55 (1.35)
Illness Duration (months), mean (SD)		16.67 (8.75)
**Sex, n (%)**		
	Male	0 (0)
	Female	9 (100)
**Highest level of education, n (%)**		
	Primary	7 (78)
	Secondary	2 (22)
	Tertiary	0 (0)
**Diagnosis, n (%)**		
	AN^a^-restrictive	8 (89)
	AN-purging	1 (11)
	BN^b^	0 (0)
	EDNOS	0 (0)
**Current treatment type, n (%)**		
	Day Hospital	1 (11)
	Outpatient	8 (89)

^a^AN: anorexia nervosa.

^b^BN: bulimia nervosa.

^c^EDNOS: eating disorder not otherwise specified.

**Table 2:** Description of eating disorder specialists.

ED specialist characteristics (N=8)		Values
Age (years), mean (SD)		34.63 (7.21)
**Sex, n (%)**		
	Male	2 (25)
	Female	6 (75)
**Specialty, n (%)**		
	Psychiatry	2 (25)
	Psychology	5 (63)
	Nursing	1 (12)
**Employment status, n (%)**		
	Public	4 (50)
	Private	4 (50)
Duration actual employment status (years), mean (SD)		6.19 (4.29)

### Focus Groups

Qualitative data were collected through four in-depth focus groups, as previously indicated. The first comprised health care providers from the COMB, the second focus group was conducted with mHealth experts from the ACES, a third focus group with patients who had used the mHealth tool, TCApp, and a fourth with ED specialists who had monitored such patients on the Web.

The interviews carried out in the first 2 focus groups included open-ended questions designed to address the following topics relating to drivers and barriers of mHealth adoption:

- Factors related to mHealth characteristics
- Individual and professional factors
- Human environment factors
- Organizational environment factors: internal and external

Open-ended questions for the focus group were designed following previous work of our team [37]. This broader study had the objective to measure ICT adoption among 25 General Practitioners, representing 20 different European countries. The framework used in this previous study integrated several behavioral models and hypotheses extracted from the international literature (ie, Technology Adoption Model and its revised version Technology Adoption Model 2, Universal Theory of Acceptance and Use of Technology, Theory of Reasoned Action, and Theory of Planned Behavior). However, when designing the questions for the first and second focus group, little was yet known about general mHealth adoption among health care professionals. Thus, we adapted questions developed in the previously mentioned study to suit mHealth characteristics*,* and we were also inspired by the adoption factors identified in the systematic review by Gagnon and colleagues [11].

For the third and the fourth focus group, the interviews with patients and ED specialists were completed 3 months after the study start date. When designing the questions, we were inspired by a multidimensional evaluation framework, called Model for ASessment of mHealth (MASH), previously described and implemented by a European project in which our team also participated, termed DoChange. The MASH model was constructed on the basis of the evaluation framework of the Model for ASsessment of Telemedicine methodology [38]. We followed 5 of the 8 domains of the MASH model, which were translated into the following research questions that guided our focus groups:

- Domain 3 (both patients and ED specialists). Effectiveness.

To what extent did patients’ ED symptoms and quality of life improve when treated with TCApp as a complement to face-to-face treatment?

- Domain 4 (both patients and ED specialists). User experiences.

Which were professionals’/patients’ needs in implementing the TCApp services in daily practice? Were users satisfied with the app/usability of the services offered by the TCApp? Which were the most important facilitators in implementing the TCApp in routine practice? Which were the most important barriers in implementing the TCApp in routine practice?

- Domain 5 (ED specialists). Economic aspects.

Are you willing to pay for use of the TCApp from now on in your routine practice?

- Domain 6 (both patients and ED specialists). Organizational aspects.

Participants had to think of a future ideal scenario in which decisions at their institution were made by them and describe the specific proposals they would make to their organization about a new service that implements, in an ideal way, the TCApp together with other new technologies.

- Domain 7 (both patients and ED specialists). Sociocultural aspects.

Have any issues arisen regarding cultural accessibility (considering the different languages spoken by the users) and socioeconomic accessibility (different ages, users’ digital health literacy)?

### Study Procedure

#### Health Care Providers and Mobile Health Experts

First, for the focus groups with health care providers and mHealth experts, a formal invitation was sent to both institutions (COMB and ACES, respectively) by the principal investigator of the study, FL. Both focus groups, of 1 hour each, were conducted by FL at the headquarters of ACES and at the UOC, and they were audio recorded with permission. A second researcher, CF, was present during these events and transcribed the group discussions verbatim.

#### Mobile Health Intervention TCApp

The study procedure of the mHealth intervention was described in detail elsewhere [36]; therefore, we will only briefly describe the important aspects of the study protocol here. After an initial semistructured interview and a baseline evaluation using self-report questionnaires at T0, participants were randomly assigned to 1 of the 2 study conditions (experimental or control group). Therapists in the participating ED units were invited to introduce to the patients of the experimental group the option of using the TCApp as a complementary tool to their standard face-to-face CBT. Each therapist was given instructions on how to use the Web-based environment intended for therapists, on how to add patients as users, and each therapist was given instructions on the basic functionalities of the app specifically designed for patients. Therapists were asked to explain briefly to each patient in the experimental group how to use the TCApp, in addition to supplying them with a brochure with written instructions, as well as an encouragement to choose a nonidentifying username. Over a 12-week period, patients were expected to use the TCApp at least once a week, completing self-records or contacting their therapist via chat on a regular basis when this was considered necessary. The therapist responsible for the Web-based monitoring was asked to, at least once a week, connect to the Web-based platform and perform the following actions: follow the patient’s daily self-records, generate personalized reports or graphs, and communicate with the patient via the chat function. Little, if any, additional instruction was given on how to use the app clinically, to allow for natural adoption by patients as well as clinicians. In turn, patients in the control group were told that access to the TCApp would be offered to them after a waiting period of 6 months. Thus, for a period of 12 weeks, each group of patients received the treatment that corresponded to the patients’ experimental condition, in addition to the patients’ regular treatment. At the end of the 12-week period, patients from the experimental group were told to stop using the TCApp (they were discharged from the service), and a posttreatment evaluation was carried out with both groups of patients (T1). Furthermore, 1 week later, an email invitation was sent to patients from the experimental group and ED specialists who had monitored their patients on the Web, asking them to participate in the present focus groups. Both focus groups with patients and ED specialists were conducted in the respective participating hospitals by DA, and both they were audio-recorded with permission. The duration of these focus groups was an hour on average per group. During the focus group with patients and directly after the group discussion, self-report questionnaires were administered, whose intended purpose was to evaluate usability of and participants’ satisfaction with the tool. Similarly, a self-report questionnaire designed by our research team was administered to ED specialists after the group interview. A researcher of our group, CF, transcribed the group discussions after listening to the audio recordings. Subsequently, DA read and validated these transcriptions.

### Additional Measures Used in the Mobile Health Intervention TCApp

#### For Patients

##### Sociodemographic and Clinical Characteristics

Patient information, including age, sex, diagnosis, illness duration, body mass index (BMI), degree of education, and employment status, was retrieved from the medical history of each patient, which was provided to the research team by the ED specialists responsible for each participating center.

##### Users’ Experience Using the TCApp

*System Usability Scale (SUS)* [39] is a 10-item questionnaire that measures usability of a range of systems and has been described elsewhere [36].

*Client Satisfaction Questionnaire (CSQ-8)* [40] is an 8-item questionnaire that is designed to measure client satisfaction with services and has been described elsewhere [36].

#### For Eating Disorder Specialists

*Sociodemographic characteristics*, including age, sex, employment status, and specialty, were collected through an introductory self-report questionnaire.

### Data Analysis

Thematic content analysis was used to analyze the qualitative data from the 4 focus group discussions. [41]. First, members of the research team (DA and FF) independently read and reread the transcripts of the 4 group discussions to familiarize themselves with the data. They then sought to achieve consensus regarding a common coding scheme for the information collected during the group discussions. Common categories of meaning in the data relating to the study objectives were identified inductively [33]. In case of doubt during coding, researchers stepped back and reclassified the coded data to ensure that any contradictory information was not omitted. After having categorized drivers and barriers of mHealth adoption, we compared our findings with the classification proposed by Gagnon and colleagues [11], as well as with previous research conducted by our team [37]. The data were coded manually, and no software or tool was used in this procedural step. Finally, the most significant and representative examples for each theme were collected from the transcripts after discussion among researchers on the team. To better reflect patient and provider experiences, all information was presented in tables. Each key theme was classified depending on how frequently it had been mentioned by participants, using +++ for the most frequently mentioned themes, ++ for frequently mentioned themes, and + for themes that were only occasionally mentioned (and sometimes generated a debate because of contradictory opinions on the topic). In addition, advantages and disadvantages of the TCApp as perceived by patients and ED specialists were also classified in common themes and domains, together with the frequency with which each theme was reported by patients and specialists (again ranking from + to +++).

The analyses of all descriptive data regarding participants’ sociodemographic and clinical characteristics, SUS, and CSQ-8 were carried out using SPSS 20.0 (SPSS Inc) [42]. Credibility and validity of patients’ responses were ensured through cross-verification (ie, triangulation) of the outcomes of the focus groups relating to usability and satisfaction and the SUS and CSQ-8 questionnaires.

## Results

### Focus Groups With Health Care Providers and Mobile Health Experts

In total, 4 domains and 16 key themes were identified and were then classified as barriers to or drivers for mHealth adoption. Most of these themes, 11 (69%), were classified by professionals as barriers, and only 5 (31%) were perceived as facilitators for mHealth adoption. The complete list of domains and themes, together with professionals’ most representative examples recounted during the focus groups, is shown in Tables 3 and 4. In the same table, we also present an analysis of the factors on the basis of the frequency with which these were reported by professionals.

**Table 3:** Barriers relating to mobile health adoption by health care providers and mobile health experts.

Domain and key themes		Examples
**External factors: organizational environment**		
	Lack of time and workload (+++^a^)	“I had no time to integrate any changes in my work schedule”; “I know the theory...that you have to do a first push at the beginning in order to integrate changes, for example change to e-consultations, and then the workload will become less. But then in practice, it is difficult to implement due to our workload.”
	Management: Lack of strategic plan to implement mobile health (+++)	“Leaders have to believe in innovation in order to promote the use of ICT^b^ tools, otherwise projects testing their efficacy will not succeed.”
	Health care policies and sociopolitical context: Lack of budget and direct interest (+++)	“Technology costs...There is a considerable lack of budget to support studies assessing the impact of mHealth tools”; “At a political and legislative level, there is not enough interest in mHealth. Doctors complain that they cannot reimburse mHealth”; “Technology is costly and it is difficult to verify its return. Often, the ideas we propose seem good, but when the budget is specified, people become more resistant, you see that the investment has a certain cost. In addition, technology evolves fast and you may not have time to recover the initial investment...Thus, inversions for mHealth should not only be innovative, but also timely and always updated.”
	Insufficient training (++^c^)	“It is important to have a properly trained team...It is not enough to have an IT-department specialised and dedicated to this. All the staff has to be properly trained”; “It is essential to offer continuous training to ensure that students of health sciences acquire digital competences.”
	Human resources: Lack of information technology support (++)	“There is a considerable lack of support by the technical staff of our institution when we integrate a new mHealth tool into our services...Due to this lack of support, users either use the new tools in the wrong way, or stop using them because they get frustrated when they do not know how to use them in a proper way.”
**Individual factors**		
	Age: Lack of familiarity and mobile health skills (+^d^)	“Age-based digital divide is present in the health sector...Young health professionals have more digital minds...Instead, for many professionals who are older in age, the handling of the Internet and other ICTs may seem complex and they prefer to do things in the traditional manner (paper-and-pencil methods)”; “I do not think that age has a significant impact on adoption...In my institution, older professionals do not have more negative attitudes towards ICTs than younger ones, neither do they perceive their utility and usefulness differently.”
	Lack of agreement with mobile health: Resistance (++)	“Professionals are often resistant to change because of fear of the unknown and new.”
	Risk-benefit assessment (perception; +)	“The value of face-to-face contact with our patients is inherent to all of us...sometimes we are afraid of losing this when introducing a new technology.”
**External factors: Human environment**		
	Insufficient interaction: Patient-Health professional-information technology team (++)	“New technologies cannot be implanted unilaterally by the IT team. Health professionals are those who really know what patients need. There has to be an alignment of needs between IT team, patients and health professionals.”
**Mobile health characteristics**		
	Security and privacy issues (++)	“Sometimes bureaucracy is used as an excuse to stop the implementation of an innovation...Once patients sign their informed concern, there is freedom to conduct innovations”; “We have a very protectionist system...The new law on security / privacy of medical histories is very restrictive about which data from patients can be viewed and shared...this also obstructs the sharing of this information online using mHealth tools”; “It is important to approve Apps by an official authority taking into account both technological validation (must be useful) and functional validation (applicable to the context).”
	Design and technical concerns (+)	“Technology can fail”...“Technical limitations of the mHealth tools”

^a^+++: Very frequently mentioned (≥4 times).

^b^ICT: Information and Communication Technology/ies.

^c^++: Frequently mentioned (2-3 times)

^d^+: Only occasionally mentioned, sometimes a debate was opened because of contradictory opinions (mentioned 1 time, or 2 contradictory opinions).

**Table 4:** Drivers relating to mobile health adoption by health care providers and mobile health experts.

Domain and key themes		Examples
**Mobile health characteristics**		
	Quality standard (+++^a^)	“It is very important to achieve a proper quality control and approval of a mHealth tool by a certified institution”
**Individual factors**		
	Economic incentives for professionals (+^b^)	“If I personally had to implement a mHealth tool as complementary to my face-to-face visits and charge each visit by 2 euros more, I would think about it”; “I would rather offer training incentives to professionals as economic incentives in the public sector are not always realistic.”
**External Factors: Human environment**		
	Support and promotion of mobile health by colleagues (++^c^)	“All stakeholders should participate in the design of the mHealth tools (usability, acceptability, feasibility).”
**External factors: organizational environment**		
	Training (+++)	“Offer continuous training...Create awareness and empowerment of patient and professional before mHealth implementation.”
	Management: Strategic plan to implement mobile health (+++)	“It’s a top-down approach...Leaders have to believe in innovation and push for its implementation.”

^a^+++: Very frequently mentioned (≥4 times).

^b^+: Only occasionally mentioned, sometimes a debate was opened because of contradictory opinions (mentioned 1 time, or 2 contradictory opinions).

^c^++: Frequently mentioned (2-3 times).

Regarding barriers, the most recurrent themes pertained to the *Organizational Environment* domain. First, the lack of time and workload were frequently mentioned by professionals as barriers (eg, *“I had no time to integrate any changes in my work schedule”).* Second, the lack of a strategic plan by leaders in health institutions to implement mHealth was also reported (eg, *“Leaders have to believe in innovation in order to promote the use of ICT tools, otherwise projects testing their efficacy will not succeed”).* Third, the lack of budget and direct interest at a legislative or political level supporting mHealth implementation was another common barrier (eg, *“Technology costs...There is a considerable lack of budget to support studies assessing the impact of mHealth tools”*). Finally, the lack of training of professionals (eg, *“It is essential to offer continuous training to ensure that students of health sciences acquire digital competences”*) and the lack of support by the information technology team in health institutions (eg, “...*Due to this lack of support, users either use the new tools in the wrong way, or stop using them because they get frustrated when they do not know how to use them in a proper way”*) were other main themes identified as barriers that belonged to the *Organizational Environment* domain, but they were less frequently mentioned by participants. Age and risk-benefit balance were identified as barriers in the *Individual* domain, and these generated diverse and sometimes contradictory opinions by professionals. For example, some of these professionals believed that a digital divide was a common problem found in health care institutions:

Young health professionals have more digital minds...Instead, for many professionals who are older in age, the handling of the Internet and other ICTs may seem complex and they prefer to do things in the traditional manner -paper-and-pencil methods

However, others thought the opposite:

In my institution, older professionals do not have more negative attitudes towards ICTs than younger ones, neither do they perceive differently their utility and usefulness

In relation to the risk-benefit balance, some professionals believed that the fear of losing face-to-face contact with patients was a reality difficult to overcome (eg, *“The value of face-to-face contact with our patients is inherent to all of us...sometimes we are afraid of losing this when introducing a new technology”*), although others did not agree. The individual resistance to implement mHealth services as a health professional was also seen as a barrier to the adoption of mHealth. In fact, many participants declared that they were resistant to change because of their fear of the unknown. The insufficient interaction among patient, health professional, and information technology team was another barrier to mHealth adoption for the domain of *External factors relating to the human environment*. For example, a health care provider reported the following:

New technologies cannot be implanted unilaterally by the IT team. Health professionals are those who really know what patients need. There has to be an alignment of needs between IT team, patients, and health professionals

Finally, security and privacy concerns, as well as design and technical issues, all of which related to *mHealth characteristics*, were less frequently mentioned and sometimes generated contradictory opinions among respondents. For example, professionals were worried about the security and confidentiality of the data transferred through these technologies, and they declared that they had to cope with a protectionist (Spanish) health care system that is very restrictive with sharing patients’ information on the Web using mHealth tools. Regarding drivers for mHealth adoption, the importance of professionals’ training first and the subsequent implementation of an mHealth-related strategic plan by leaders were the most recurrent themes pertaining to the *Organizational Environment* domain. For example, most professionals highlighted the importance of a continuous training offered to them by their institution so that they could become more knowledgeable and empowered before using an mHealth tool in their regular visits. Next, the importance of having leaders who believe in mHealth innovations was very frequently mentioned as a driver for adoption (eg, *“It’s a top-down approach...Leaders have to believe in innovation and push for its implementation”*).

Moreover, the option to offer economic incentives to professionals (eg, *“If I personally had to implement a mHealth tool as complementary to my face-to-face visits and charge each visit 2 euros more, I would think about it”*) was an individual driver that generated contradictory opinions, as it could be counterproductive at times (eg, *“I would rather offer training incentives to professionals, as economic incentives in the public sector are not always realistic”*). Finally, the importance of submitting an app to quality control to get proper homologation before implementation in clinical practice was another theme relating to mHealth characteristics, which was frequently mentioned as a driver.

### Mobile Health Intervention Study

First, as regards patients’ scores on the 2 self-report questionnaires administered after the group interview, the mean of the total CSQ-8 scale for the 9 patients was 16 (SD 4.69; Range=11-22), whereas the mean score for the SUS scale was 81.56 (SD 22.52; Range=27.50-100). This indicated moderate usability of and high satisfaction of patients with the tool. Tables 5 and 6 summarize qualitative information from the posttreatment focus group for participants in the experimental group.

**Table 5:** Perceived advantages of the TCApp by patients.

Domain and Key themes		Patients who agree with example statements (N=9), n	Examples of patients´ statements
**Mobile health characteristics**		
	Perceived ease of use	9	“At the beginning I needed some instructions and guidance but then it was very easy to use.”
	Perceived usefulness	8	“Paper food records are a source of discomfort because they can be lost or forgotten at home while online records are comfortable and useful”; “The option of taking photos of your meals and send them to your therapist was very useful”; “I would recommend the app to a friend with a similar problem.”
	Design: App	9	“I found the online platform very attractive”; “I liked the colours and the personalized avatar.”
	Satisfaction with content available: Motivational components	2	“I liked the option that we had to receive rewards a lot...I was looking forward to receiving prizes depending on my weekly performance and to comparing my ranking with others.”
	Content appropriate for users (relevance)	6	“The app facilitated a better understanding of the contextual variables that surrounded my eating behavior...I am now more aware of what happens before, during and after the problematic behavior I would like to change...more than I used to be with paper records”; “It is a good company during your treatment process, especially when you feel lonely or with the urge to carry out a problematic behavior...The option to share your thoughts, emotions or actions and be sure that your therapist is going to read them, relieves stress and guilt.”

**Table 6:** Perceived disadvantages of the TCApp by patients.

Domain and Key themes		Patients who agree with example statements (N=9), n	Examples of patients´ statements
**Mobile health characteristics**		
	Privacy and anonymity concerns	1	“Sometimes I did not know who was going to read my messages, something that stopped me from using the chat option.”
	Negative perception of usefulness	2	“It is not always useful to keep track of your problematic behavior and quantify it...I would have liked it more if the app had offered a free text option next to each question asking for the presence and frequency of several symptoms (vomiting, restriction, intensive exercise, laxative use, etc.) so that we could have the opportunity to write further explanations or observations of our behaviors.”
	Problems with the design: App	9	“There was a word limit when I was using the chat, I could not finish my messages so I was sending them incomplete to my online therapist or split into 2 or 3 different messages”; “There was a 24-hour limit in the app; this means that you could not register your activity after 24.00 PM...that appeared as activity of the following day”; “I would prefer if the app collected information retrospectively, meaning that the day after you register what you did the day before. By doing so, you can gain a better insight of your behavior”; “It would have been nicer to change the design of the chat (each message as a new e-mail in your inbox) and make it similar to the chat of Facebook or Whatsapp, in order to see the whole history of conversations with your therapist or be able to see when he/she reads your messages, etc.”
	Content inappropriate for users	3	“Patients receiving intensive treatment, i.e. day hospital, have enough of support and don’t want more monitoring of their symptoms. The content of the app is not appropriate for their needs. Then, as regards outpatients, when they are asked to complete self-records daily, they feel as if they retrocede in their treatment process. The app is not useful for them either”; “I think the app can be more appropriate for patients at their early treatment stages, when they are expected to gain awareness of their ED-related behaviors.”
	Lack of satisfaction with content available: Lack of personalization	9	“It would have been better to personalize the app according to ED diagnosis, type of treatment that each patient is receiving and also treatment stage.”
**External factors: Human environment**		
	Patient and therapist limited and not-personalized interaction	3	“The professional who was following me online was not the same with the one with whom a had face-to-face sessions...Sometimes I perceived a lack of understanding of my problems by my online therapist”; “Sometimes it took him/her a lot to answer to my online messages and when I finally received an answer, this was no longer useful to me”; “I would like to receive more immediate and personalized answers to my messages.”
**External factors: Organizational environment**		
	Study design: Strict instructions	1	“There was a lot of pressure by researchers to use the app once a day, even if it was not always necessary.”
**Individual factors**		
	Negative benefit-risk balance	1	“It was easier for me to lie through the online records compared to the paper self-records...The app is more private, nobody (referring to her parents) has access to your records.”

As regards patients’ perceptions about *usability* of the TCApp, all participants (9/9) perceived that the app was easy to use and very practical. Participants were comfortable with the app, and none of them neither reported discomfort with the technology nor were they faced with technical problems. At the start of the intervention, some assistance was required by all participants to ensure that the app was working properly. Regarding *usefulness*, most participants (8/9) indicated that the app was very useful for the time between regular visits to health care professionals, and they asserted that they would recommend the tool to a friend faced with a similar problem. Most participants (8/9) agreed that keeping food records, which they used to track manually in their standard CBT, generally posed a source of discomfort, as they could be lost or forgotten at home, and most participants agreed that a change to Web-based food records was more comfortable and easy to fill in on a daily basis. A total of 2 participants voiced negative comments concerning the usefulness of the TCApp, which were related to the fact that quantification of ED symptoms through presence or absence option was not always useful for them. Instead, more *free text* options could have given patients the opportunity to describe the context around symptoms and their function more comprehensively. Several problems regarding the *design* of the app were reported by patients (9/9), including problems with word limit, a 24-hour time limit (ie, entries for a day could only be entered on that particular day’s 24-hour window and not the next day), or concerns regarding the design of the Web-based chat with the therapist, which used a rather awkward and unhelpful format (see patients’ examples in Tables 5 and 6). In addition, 1 patient reported that the single option of collecting data in real time was not always convenient and helpful, given that patients were already struggling with negative feelings concerning their problematic behavior. Having to conduct another task at the same time made the experience even more difficult. However, positive comments were also raised by patients regarding the design of the app, with all participants endorsing it for its visual attractiveness and possibility to personalize settings. With regard to patients’ *satisfaction with the TCApp’s content*, the views differed. On the one hand, only 2 patients appreciated the rewards and prizes received and felt that this technique increased their motivation to keep engaged with the app. On the other hand, all patients viewed the lack of personalization of the tool according to their specific needs rather negatively (ie, type of diagnosis, type of treatment received, and individual readiness to change). Regarding the *appropriateness of the TCApp’s content,* opinions among participants varied as well. Positive comments expressed by 6 patients included the ability of the app to make patients gain a better understanding of their problematic behaviors. In addition, the app was described as a good companion that helped patients to better manage their negative feelings. A patient specifically stated the following:

It is a good company during your treatment process, especially when you feel lonely or with the urge to carry out a problematic behavior. The option to share your thoughts, emotions or actions, and be sure that your therapist is going to read them, relieves stress and guilt

On the other hand, negative comments (3 out of 9 patients) included the inability of the app to satisfy treatment needs of day-hospital patients and outpatients (see patients’ examples in Tables 5 and 6). A total of 2 important issues were raised by 3 patients of 2 public institutions who seemed to encounter problems with fully complying with the study protocol. The first problem was related to *privacy and anonymity concerns:* Although patients were expecting to be monitored on the Web by the same therapist who was following them in their regular face-to-face visits, this was not always possible in the participating hospitals because of the work burden with which therapists were faced. As a consequence, messages were often read and answered by another therapist, who had no knowledge of the patient’s clinical history. This was reason for some concern with certain patients regarding the privacy of their personal information and their expectation and desire to feel more secure when using the app. The second issue concerned the *limited and not-personalized patient-therapist interaction.* All 3 patients indicated that therapists at times did not answer their Web-based messages immediately and that when they did, answers were rather impersonal. As a result, these patients perceived a lack of guidance in their treatment when using the chat option. Finally, another negative comment expressed by 1 patient was related to organizational factors; specifically, it was related to the strict instructions given by the research team to follow the *study design*.

Tables 7 and 8 summarize findings from the posttreatment focus group with eating disorder specialists who monitored patients on the Web.

**Table 7:** Perceived advantages of the TCApp by eating disorder specialists.

Domain and Key themes		ED specialists who agree with example statements (N=8), n	Examples of ED specialists’ statements
**Mobile health characteristics**		
	Perceived ease of use	8	“The platform (for professionals) is easy to use, very practical, quick and intuitive”; “Patients found it (TCApp) easy to use, simple, and they learned fast how to use its different functionalities. They have never asked for more explanations than those given at the beginning."
	Perceived usefulness	8	“I value the immediacy of the instrument and the ability to advance visits when things were not going well a lot...I believe that the app can be a good tool for therapists. They can have information prior to their face-to-face visits.”; “The app facilitates our clinical practice a lot...The whole team feels more reassured with regard to each patient´s treatment”; “I value the possibility to give patients quick and valuable information (mostly resolve their doubts) during the time in between their regular sessions or advance visits, when there is something worrying in their records or messages...By doing so, we can also reduce the amount of visits of our patients to the emergency department”; “The online chat provides very valuable information as it facilitates contact with patients in-between sessions. However, it does require more time to explore and exploit the potential of these messages and how to use the information provided by each patient in face-to-face sessions.”
	Design: Platform	8	“The platform is attractive and does not overload visually”; “The possibility to receive photographs of the patients’ meals is great!! I like how the food records described with words appear in the same screen next to the meal photographs.”

**Table 8:** Perceived disadvantages of the TCApp by eating disorder specialists.

Domain and Key themes		ED specialists who agree with example statements (N=8), n	Examples of ED specialists´ statements
**Mobile health characteristics**		
	Problems with the design: Platform	8	“A different new screen was opened for each of my patients...It was difficult for me to follow several patients at the same time.”
	Technical problems	1	“In some cases, the application stopped working; this meant a lot of extra work without getting good results. Surely it depended on a user‘s smartphone model (Chinese), or it occurred due to the fact that they had downloaded another application that was interfering with the use of the TCApp.”
	Lack of satisfaction with content available I: Inappropriate motivational components	2	“I would modify all content (she refers to the option offered to patients by the app to receive prizes and rewards according to their performance). Many patients with AN profile are stressed when receiving prizes; they want to be first in the list, corresponding to their high levels of perfectionism and competitiveness. It can be counterproductive for these patients”; “My patients decided not to share the awards received through social networks for confidentiality issues. They wanted to keep everything inside the application and not get out of this environment.”
	Lack of satisfaction with content available II: Lack of personalization	8	“The app should be personalised according to patients’ clinical characteristics...for example, severe patients who presented a lack of consistency in their treatment in general, showed the same with the app”; “You cannot use the same instructions concerning the frequency of use of the app (once a day) for all patients. You should assess before whether this frequency is beneficial for them or not, according to the treatment stage, specific diagnosis, and other clinical characteristics of your patients”;“We should try to accommodate the therapeutic objectives offered by the app to the patient’s profile.”
	Lack of satisfaction with content available III: Monotonous activity	1	“Patients’ online activity was monotonous...for example, emotions experienced during the day were static...a specific emotion felt at a specific moment during the day cannot represent the emotional status of the patient for the entire day...Some further specifications may be added, for example, next to each emotion, an objective for the following week or a further explanation of the context around emotions and their function could be provided, in order to make emotion records more motivating for them.”
	Lack of satisfaction with content available IV: App was not interactive	3	“The app was not dynamic and interactive enough and its functionalities depended on the initiative demonstrated by the patient. Therefore, at times, when patients were feeling worse, they had no initiative to interact actively with the app and thus, they did not use it at all”; “The use of the app depended exclusively on each patient. For the most resistant and difficult patients, we had to follow-up a lot and remind them during sessions that they had to use the app regularly.”
**External factors: Human environment**		
	Lack of appropriate collaboration among colleagues	1	“Lack of coordination among different health professionals participating in the study. Sometimes it was not clear who was doing what.”
**External factors: Organizational environment**		
	Study design: Strict instructions	1	“Some of my patients felt frustrated and under pressure with the fact that they had to use the app at least once a day.”
	Economic resources available	2	“In private institutions (ie, Dexeus), sometimes patients are willing to replace face-to-face sessions with their therapist with the chat option offered by the app There are economic reasons behind this...I think that in private hospitals, the app should be used differently; you cannot force a patient to have extra visits if you consider it important, as you would do in a public hospital”; “The implementation of the app in private practice is easier...You can charge each visit 2 euros more, for example, and offer patients complementary treatment with the app...In public hospitals this option does not exist at the moment, as there is a lack of budget and of direct interest in mHealth.”
	Lack of time and workload	6	“Due to workload or need to attend emergencies and other priorities, sometimes patient monitoring through the app was postponed”; “Unfortunately, sometimes the symptom monitoring was not regular and constant (by us), as initially agreed when we were given the study protocol.”

All of the clinicians reported that the TCApp was helpful in their treatment, and all preferred this method to keeping paper records. In terms of *ease of use*, all clinicians agreed that the app was practical, quick, and intuitive; instructions were clear, and no further technical support was needed. In terms of *usefulness*, comments were positive and mostly focused on the usefulness of the Web-based chat function. Clinicians valued the immediacy of the instrument and the possibility to anticipate visits when necessary or to use in standard therapy all the valuable information provided by the app regarding the patient’s week. In the long run, it is mentioned that the use of the app can also be cost-effective, as it can reduce patient’s visits to emergency departments. A therapist specifically stated the following:

The app facilitates our clinical practice a lot...The whole team feels more reassured with regard to each patient´s treatment.

The *design of the Web-based platform* was attractive and not visually overloading, and all therapists positively valued the option to receive photographs of their patients’ meals.

The most frequently reported disadvantages of the app concerned (1) the lack of personalization of the app, (2) some problems with the design of the platform, and (3) the lack of time to regularly monitor patients because of workload. First, regarding the *lack of personalization of the app*, all therapists agreed that the app should be personalized in the future according to patients’ clinical characteristics and specific needs (ie, sex, age, living with caregiver, treatment type, ED profile, and private vs public institution). A therapist also proposed to validate a protocol of correct use of the app according to a patient’s profile, specifying the frequency of use of the app and possible functionalities relevant for a specific group of patients. Second, the only problem with the *design of the platform* concerned the difficulty expressed by therapists to follow several patients at the same time, given that a new screen was opened for each patient. Third, most of the therapists (6/8) stated that they could not always comply with the study protocol (monitor each patient on the Web at least once a week) because of their *workload* or having to attend to emergencies and other priorities. A total of 3 out of 8 therapists stated that the *app was not interactive* enough and that the use of each functionality (food records, emotion/thought/action records, chat, etc) depended on patients’ self-determination and willingness to use the tool. A therapist commented on this saying the following:

The use of the app depended exclusively on each patient. For the most resistant and difficult patients, we had to follow-up a lot and remind them during sessions that they had to use the app regularly.

Contradictory opinions existed regarding whether the app would be more easily implemented in private institutions or public institutions. The fear of replacing face-to-face sessions by the chat option, which is of course less costly, was expressed by a therapist working in the private sector. Another therapist from a public hospital said that the implementation of the app should be easier in private practice, as there are more economic recourses available for the integration of a complementary service that includes the TCApp. The *inappropriateness of the motivational components* employed by the TCApp (rewards, prizes, and ranking of users according to their performance) for patients with EDs, especially those with a restrictive profile, was mentioned by 2 therapists. One of them asserted the following:

I would modify all their content...Many patients with AN profile are stressed when receiving prizes; they want to be first in the list, corresponding to their high levels of perfectionism and competitiveness. It can be counterproductive for these patients.

Finally, other disadvantages related to mHealth characteristics and the organizational and human environment shown in Tables 7 and 8 were reported by therapists only once. Regarding future modifications and points for improvement, the following topics were discussed:

- Personalize the app according to patients’ needs and specific characteristics.
- Offer retrospective record keeping of the problematic behavior, in conjunction with real-time records. For example, a therapist commented on this saying the following:

Some of my patients would have preferred it if they had had the opportunity to register a problematic behavior (restriction, vomit, binge...) the day after its occurrence. Many of the patients from the control group that normally used paper records filled the record the day after the event or when they were feeling calmer or more distanced from the problematic behavior.

- Adapt the TCApp to nutritionists’ needs. A therapist said the following:

I think the use of the app could be more appropriate for nutritionists and not for psychologists...an additional functionality assessing the caloric content of the meal records may be an option.

- Add psychoeducational material and relaxation or mindfulness techniques in the form of modules that would be activated according to patients’ profile and evolution. Improve the gamified environment to make the app more interactive and motivating, for example, by including vodcasts with motivational messages by recovered patients or messages by therapists, personalized reminders, objectives, or coping strategies close to each problematic behavior.

## Discussion

### Principal Findings

Emerging literature on the field has shown that app-based treatment suggests promising results for enhancing the quality of mental health provision and treatment outcomes, whereas, at the same time, it can improve engagement with respect to different mental disorders, including depression, anxiety, stress, substance use, and symptoms of EDs [43,44]. Despite reported advantages for patients and health care providers, a large group of health professionals still seems somewhat critical and reluctant in the adoption of such techniques [10]. In this study, we first focused on establishing the drivers and barriers for adoption of mHealth techniques in general health care delivery processes. Second, we examined the adoption of mHealth techniques in an intervention for patients with EDs and their therapists.

The results showed that in the focus groups with health care providers and mHealth experts, most of the recurrent themes were classified as barriers, less so as facilitators, for mHealth adoption. This indicates that most professionals considered mHealth techniques as difficult to obtain and use. In addition, most barriers were attributed to external factors relating to the human or organizational environment rather than internal factors relating to individual, personal obstacles. More specifically, most health professionals reported a lack of time because of workload, a lack of strategic plan by leaders, lack of budget, and direct interest at a legislative or political level to support the implementation of mHealth. Other external barriers, which were less frequently reported, concerned the lack of specific ICT training for health professionals and the lack of communication and support among patients, health professionals, and information technology teams. In addition, individual factors, including the age-related digital divide and the fear of losing face-to-face contact with patients, generated contradictory opinions among professionals. These findings are in line with previous studies [11,13], suggesting that health care providers are still somewhat resistant and conservative about integrating mHealth technologies in their daily practice. If they decide to adopt such tools, they report that they need to feel skilled, trained, and supported by the IT team and the leaders of their institution in an adequate fashion. Similarly, Lindgreen and colleagues [30] have shown that clinicians in such situations were primarily preoccupied with challenges that related to workload. For example, some of the clinicians in the study reported frustration because of the fact that they were not supposed to monitor patient app data outside office hours, and they expressed concerns regarding the deterioration of the patient-clinician relationship. In addition, an undesired consequence of adopting mHealth techniques may be reduced clinician work satisfaction—particularly when their technological self-efficacy levels are not considered and addressed through educational and training efforts where necessary.

The results of the mHealth intervention study indicate that the TCApp was considered easy to use and useful, although patients and ED specialists monitoring patients on the Web reported different problems in adoption. Patients valued as highly positive the Web-based food records and the role these played in helping them gain a better understanding of their symptoms. In contrast, the problems reported most frequently concerned the lack of personalization of the app according to their needs (eg, diagnosis, type, and stage of treatment), as well as the overwhelming quantification of symptoms through the app (eg, presence or absence options) instead of a more qualitative evaluation of them on the basis of cognitive-behavioral chain analyses through Web-based free-text options. Similarly, a previous qualitative study with focus groups by Juarascio and colleagues [28] for patients diagnosed with binge eating indicated that the app used (including self-help material, behavior monitoring, and provisions of real-time interventions) was deemed workable and acceptable by both patients and clinicians as a complementary tool to regular treatment, although concerns were expressed about the degree of personalization and customizability of the tool. Similarly, Darcy and colleagues [27] reported that a simple self-monitoring app based on the CBT principles for ED patients was feasible and acceptable to both patients and clinicians. Last but not least, the qualitative study by Basterfield and colleagues [45] explored how individuals with an ED used various forms of technology, and the study highlighted the need for personalization, convenience, and easy-to-follow design. Participants also expressed concerns regarding safety of ICT tools, including the presence of triggering Web-based material.

In turn, ED specialists in this study highly appreciated the Web-based environment that was offered through the TCApp, especially the Web-based chat option, the usefulness of the tool outside and inside the therapeutic context, its immediacy, and the possibility to prepare for visits in advance when they felt this to be necessary. This partly runs counter to results found in the short message service text messaging intervention by Mazzeo and colleagues [46], targeting adolescent patients diagnosed with binge eating. Although their intervention showed good feasibility as reported by therapists, the adolescents in the study expressed a lack of enthusiasm for the texting component of the intervention. These findings were rather surprising, given the positive results of other studies that have used text messaging as an element of Web-based treatment for bulimia nervosa (BN) [47] and anorexia nervosa (AN) [48,49].

Both patients and health professionals of the mHealth intervention study experienced problems in complying with the study protocol at times. One of the reasons for this was the fact that patients, mostly those who were already receiving a demanding treatment at their hospital (day hospital), felt overloaded with the Web-based tasks that they had to perform on a daily basis. Another problem was that ED specialists, mostly those working in public hospitals, because of their workload or having to attend to emergencies and other priorities, tended to not give immediate responses to their patients’ Web-based messages. In fact, such responses were sometimes provided by other professionals who were available at that particular point in time, raising privacy and confidentiality issues with their patients. This somewhat contradicts the results found in the systematic review by Dowling and Rickwood [50] regarding counseling and therapy using Web-based chat, where it was shown that patients were satisfied and valued the anonymity and invisibility that could be positively gained through Web-based textual conversations.

### Strengths and Limitations

One of the main strengths of this study is that we investigated how both health professionals and patients experience mHealth techniques, as well as what they consider important factors that need improving to use such techniques more frequently. Second, we focused this study on a heterogeneous sample of health care providers and mHealth experts who deal with health-related problems on a daily basis and who could benefit from mHealth techniques extensively. Selecting a group of professionals with such diverse backgrounds, we were able to obtain more generic views of mHealth adoption issues. In addition, by focusing on a specific mental health problem, ED, we were able to examine attitudes and ramifications of both patients and ED specialists regarding the implementation of a specific mHealth tool in their daily practice. As a result, we were able to gather a broad view of mHealth adoption issues among public and private health institutions in Spain for this type of condition.

The first limitation of this study is that there seemed to be a notable homogeneity among our sample of patients in terms of clinical representation, age, and gender. In fact, only female outpatients with AN decided to participate in the focus group, all of whom were adolescents and presented good adherence to face-to-face treatment (this can be also noticed by taking a look at the almost normal BMI of the participants). The impact of the exclusively AN sample on our findings may be that this group of patients, characterized by rigidity, compulsivity, and focus on detail [51], may find the app more attractive and easy to use as a complementary tool to their regular psychological treatment than patients with other ED profiles. For example, BN patients, who are often characterized as showing mood instability and lower compliance to therapeutic tasks compared with patients with AN, could possibly demonstrate worse adherence to the additional tasks offered to them through the app. The inclusion of such patients could thus pose new challenges on how to improve the gamified environment, to get such patients more engaged in Web-based treatment. Therefore, our app does not seem to have the same transdiagnostic utility as found in a study examining the utility of another mobile phone app for EDs [27]. In addition, this study’s results can neither be generalized to an adult population nor to patients receiving more intensive types of treatment, such as hospitalization or admission to day hospital. A second limitation concerned the degree to which patients were satisfied with the tool and the trial in general, which largely depended on the institution in which the trial took place and the time and effort invested by professionals in complying with the study protocol. Those patients who indicated being most satisfied all hailed from public institutions, and the same therapists who followed patients face-to-face also followed them on the Web. In turn, the least satisfied users were also patients in public institutions, but here, because of workload, the task to monitor patients on the Web was carried out by an external psychologist (a collaborator in the study) who did not see patients in face-to-face sessions, and thus the external psychologist had limited knowledge regarding the clinical history of each patient. In addition, the response rate for the focus groups was lower than desirable, despite the various efforts to achieve a larger and a more representative sample. Finally, regarding data analysis and interpretation of this study’s findings, the way to decide whether a finding was deemed important for participants was determined purely by frequency, that is, the number of times a theme was mentioned. However, it is possible that these themes were mentioned more often not because of a high degree of importance but because of an existing social pressure to discuss a certain theme more often than other themes. Results based on the method of categorization thus applied should therefore be interpreted with some caution.

### Future Recommendations

In terms of future recommendations, all organizational, technological, and individual levels mentioned appear important for a correct mHealth implementation in clinical practice. For the individual aspect, hospitals may need to provide incentives and continuous training to encourage professionals to integrate mHealth techniques when carrying out their regular tasks. If professionals feel more empowered, skilled, and supported by their institution in adopting new technologies, they should be more willing to make use of the opportunity to try new innovations.

At an organizational level, there is a need for a strategic plan that establishes a common framework for evaluating mobile mental health apps, which allows clinicians and patients (and importantly, not only information technology teams) to identify and choose among high quality and safe mobile phone apps in accordance with their needs. Although a great number of mental health apps are readily available, and there seems to be a major potential for such apps in psychiatric assessment and interventions [14,16,32,52,53], there is limited data on their efficacy and clinical utility, and little is currently known regarding their digital security [54]. As a result, clinicians and patients remain concerned about both efficacy and privacy issues. In particular, guidelines should be established for the correct use of the TCApp by both patients and professionals, and respective functionalities should be put in place in line with each patient’s clinical profile and readiness to change, as well as professional’s needs, which was also suggested by Lindgreen and colleagues [29,30].

As regards mHealth characteristics, ED specialists recommended that the TCapp should be better personalized according to a patient’s clinical profile and that its gamified environment should be improved by integrating more useful motivational and interactive components. It was suggested that doing so would lead to improvements in engagement for the most difficult patients. A relapse prevention module should also be integrated into the TCApp for almost recovered patients who generally receive less regular visits to the hospital and who could benefit from some functionalities of the app (ie, online chat, symptoms monitoring). Other ideas for future improvement included the possibility to adapt the TCApp to nutritionists’ needs, add psychoeducational material, add relaxation or mindfulness techniques, and add a group chat functionality with a therapist who coordinates the group conversation. Last but not least, taking into account that family therapy for children and young people is the gold-standard treatment for AN [23], a version of the app should be developed for families, and its efficacy should by tested in another RCT. In fact, in some ED units that participated in the trial, it was predominantly the parents of patients who were filed in the food records that were part of their children’s treatment on a daily basis.

### Conclusions

In sum, this study shows that health professionals and patients foresee some issues that need to be resolved to increase adoption and usage of mHealth techniques in the near future. Owing to the possible benefits and cost-saving opportunities of mHealth techniques in health care [16], possibilities to overcome the barriers perceived by health professionals and patients are nevertheless extremely relevant. Last but not least, the results obtained by this study indicate that blended treatments might offer a good solution for the treatment of patients with EDs.

## Abbreviations

ACES: Associació Catalana d‘Entitats de Salut
AN: anorexia nervosa
BMI: body mass index
BN: bulimia nervosa
CBT: cognitive behavioral therapy
COMB: Colegio Oficial de Médicos de Barcelona
CSQ-8: Client Satisfaction Questionnaire
ED: eating disorder
ICT: information and communication technology
MASH: Model for ASessment of mHealth
mHealth: mobile health
RCT: randomized controlled trial
SUS: System Usability Scale
UOC: Universitat Oberta de Catalunya
